# Use of Aloes

**Published:** 1890-08-30

**Authors:** 


### USE OF ALOES.

It has long been an article of professional faith that aloes aggravates, or even causes, hæmorrhoids. Most of the old writers, and some of the new ones, tell us so. The injurious action attributed to aloes has, however, been considerably questioned of late. For instance, a recent author observes: "Aloes is very useful for fœcal sluggishness, especially in elderly people, and may be taken frequently in proper doses. . . . The thing is to find out the quantity which will produce a daily motion." Still more recently, in a paper on "Constipation," in the *Philadelphia Therapeutic Gazette*, Dr. C. L. Dodge, of Kingston, New York, says: "Of all remedies for the cure of constipation, aloes deserves the first rank." He has prescribed aloes for patients suffering from constipation and piles over and over again, and has never known of any unpleasant effects following its use. Given in proper amount, it is by far the best remedy that we possess for hæmorrhoids associated with constipation, but the dose is important. The amount usually advised in text-books and pharmacopœias is too large. More than one grain should not be given, afterwards to be reduced to half, or a quarter of a grain. But Dr. Dodge prefers aloin in 1-10 grain doses three times a day after meals; afterwards reducing the dose to twice daily. This may be continued for several weeks or months. There are several combinations of aloin which are extensively used. The following is a favourite formula: Aloin, one-fifth of a grain; belladonna, one-eighth of a grain; strychnine, one-sixtieth of a grain. But Dr. Dodge has ceased to employ anything except the aloin itself. He remarks that the formula as above is open to objections. Strychnine in this amount, limited to a single dose, can have little, if any, effect on the peristaltic action of the bowels, and the same may be said of one-eighth of a grain of belladonna. Further, if the formula is taken *ad libitum* toxic effects may be produced. Nevertheless, we think the formula is a good one, but, of course, patients must be cautioned against an *ad libitum* use. In the treatment of constipation, however, it is well to remember that insufficient supply of water, or of succulent food, is the cause of many cases of ordinary constipation.

